# Quality-of-life impact of interstitial cystitis and other pelvic pain syndromes

**DOI:** 10.3389/fpain.2023.1149783

**Published:** 2023-05-25

**Authors:** Andrew R. Cunningham, Lin Gu, Alexandra Dubinskaya, Amanda M. De Hoedt, Kamil E. Barbour, Jayoung Kim, Stephen J. Freedland, Jennifer T. Anger

**Affiliations:** ^1^ Brody School of Medicine, East Carolina University, Greenville, NC, United States; ^2^ Durham VA Health Care System, Veterans Health Administration, United States Department of Veterans Affairs, Durham, NC, United States; ^3^ Department of Urology, Cedars Sinai Medical Center, Los Angeles, CA, United States; ^4^ Division of Population Health, Centers for Disease Control and Prevention (CDC), Atlanta, GA, United States; ^5^ Center for Integrated Research on Cancer and Lifestyle, Cedars Sinai Medical Center, Los Angeles, CA, United States; ^6^ Department of Urology, University of California, San Diego, La Jolla, CA, United States

**Keywords:** intersticial cystitis, bladder pain syndrome, IC/BPS, pelvic pain, veterans

## Abstract

**Objective:**

To compare health-related quality of life (HRQOL) and pelvic pain levels over time in patients with interstitial cystitis/bladder pain syndrome (IC/BPS) and those with other pelvic pain conditions (OPPC) including chronic prostatitis, dyspareunia, vaginismus, vulvodynia, and vulvar vestibulitis.

**Methods:**

We prospectively enrolled male and female patients from any Veterans Health Administration (VHA) center in the US. They completed the Genitourinary Pain Index (GUPI) quantifying urologic HRQOL and the 12-Item Short Form Survey version 2 (SF-12) quantifying general HRQOL at enrollment and 1 year later. Participants were classified by ICD diagnosis codes and confirmed by chart review to be IC/BPS or OPPC (308 and 85 patients respectively).

**Results:**

At baseline and follow-up, IC/BPS patients, on average, had worse urologic and general HRQOL than OPPC patients. IC/BPS patients demonstrated improvement in urologic HRQOL measures over the study but demonstrated no significant change in any general HRQOL measure suggesting a condition-specific impact. Patients with OPPC demonstrated similar improvements in urologic HRQOL but had deteriorating mental health and general HRQOL at follow-up suggesting a wider general HRQOL impact for these diseases.

**Conclusions:**

We found that patients with IC/BPS had worse urologic HRQOL compared to other pelvic conditions. Despite this, IC/BPS showed stable general HRQOL over time, suggesting a more condition-specific impact on HRQOL. OPPC patients showed deteriorating general HRQOL, suggesting more widespread pain symptoms in these conditions.

## Introduction

Interstitial cystitis/bladder pain syndrome (IC/BPS) is a chronic pelvic pain condition characterized by bladder-centric pain in the absence of infection or other disease. Other pelvic pain conditions (OPPC) such as chronic prostatitis, dyspareunia, vaginismus, vulvodynia, and vulvar vestibulitis can present with similar symptoms to IC/BPS, often leading to a delay in diagnosis for all these conditions. While urine cultures, cystoscopy, and urodynamics may be useful adjuncts, they cannot be used to definitively diagnose IC/BPS (1). According to the American Urological Association, the lack of definitive testing has made IC/BPS diagnosis subjective, relying on the clinician's ability to differentiate IC/BPS from OPPC (1, 2). Thus, while the impact of IC/BPS on health-related quality of life (HRQOL) is immense (3), it has not been directly compared to that of other pelvic pain disorders. The comparative impact of IC/BPS on HRQOL in relation to other pelvic pain syndromes has not been extensively studied due to the difficulty in differentiating these conditions and lack of prospective studies identifying longitudinal changes in these conditions over time. In addition, the differential impact of these conditions on disease specific versus general HRQOL has yet to be elucidated.

We sought to address these gaps by comparing both general health-related quality of life (HRQOL) and condition specific-HRQOL prospectively over a one-year period in IC/BPS patients and those with other pelvic pain conditions (OPPC). The heterogenous population of male and female veterans nationwide provides a representative sample of U.S. patients experiencing a wide range of pelvic pain conditions. General measures of HRQOL provide data across different conditions and encompassing the entirety of a patient's condition. Evaluation of a patient's chronic conditions, comorbidities and overall health can be evaluated with general measures. Specific measures of urologic HRQOL can be utilized to identify disease-specific symptoms and measure effectiveness of interventions and treatments related to the disease. Using these two types of HRQOL measures, the Multidisciplinary Approach to the Study of Chronic Pelvic Pain (MAPP) network has acknowledged that IC/BPS can have a greater condition-specific impact on urologic HRQOL than on general HRQOL. The MAPP Network also recognized an association between chronic pelvic pain syndromes and psychosocial factors (e.g., depression) that can impact general HRQOL (4, 5). Therefore, we hypothesized that the IC/BPS cohort would demonstrate improvements in specific urologic HRQOL measures while both condition-specific and general HRQOL would improve in patients with OPPCs as symptoms had a wider impact on all HRQOL measures (specific and general) and diagnosis/treatment are generally more definitive than in IC/BPS.

## Materials and methods

### Data source/ascertainment of study cohort

Within the nation's largest nationally integrated health care system, the Veterans Health Administration (VHA), we recruited veterans nationwide into cohorts of subjects with IC/BPS and OPPC. Data from all VHA sites are collated into a common electronic medical record that is accessed through the VA Informatics and Computing Infrastructure (VINCI). We identified VA patients by querying ICD-9/10 codes (Appendix 1) and categorizing patients into IC/BPS and OPPC cohorts after detailed chart review. The IC/BPS cohort consisted of patients with confirmed chart diagnosis of IC/BPS (bladder-centric pain on two different visits at least 6 weeks apart), while the OPPC cohort consisted of patients with confirmed chart diagnosis of chronic prostatitis, dyspareunia, vaginismus, vulvodynia, or vulvar vestibulitis.

Eligible patients were sent interest and consent paperwork through the mail in accordance with IRB approved protocols, and staff followed up over the phone to obtain informed, written consent to participate in the study. After informed consent was obtained, the following study questionnaires were sent to participants at baseline and one year follow-up with responses collected via return mail:

*Female and Male Genitourinary Pain Index (GUPI)* (6)*:* The GUPI is validated for use in both men and women to quantitatively assess symptoms associated with genitourinary pain complaints. The GUPI is statistically validated to discriminate between IC, incontinence, and other pelvic conditions in both men and women while remaining highly responsive to change over time. There are three sub-scores composed of pain symptoms (score range 0–23), urinary symptoms (score range 0–10), and urinary QOL (score range 0–12) resulting in a total GUPI score of 0–45. Urologic HRQOL can be quantified by the total GUPI score, quantifying the total impacts of pain, urinary symptoms, and QOL. Sub-scores and total scores were treated as continuous variables. GUPI is used to quantify condition specific HRQOL and symptom severity. Scores were reversed from standard scale so higher score indicates better health outcomes.
*12 Item Short-Form Survey Version 2 (SF-12)* (7)*:* The SF-12 questionnaire was developed by scoring 12 items from the Medical Outcomes Study 36-Item Short-Form Health Survey (SF-36) to create a shorter questionnaire for scoring general HRQOL. The SF-12 was statistically validated for both Physical Components and Mental Components when cross-validated with the SF-36 questionnaire. The SF12 has two summary component scores: a Physical Component Summary Score and a Mental Health Component Summary Score. Scores range from 0 to 100, where zero indicates the lowest QOL and 100 indicates the highest QOL.

### Statistical analysis

The IC/BPS and OPPC cohorts' demographic and clinical characteristics at enrollment were compared using Chi-squared tests for categorical variables and *t*-tests for continuous variables. The Wilcoxon signed-rank test was used to evaluate the changes in GUPI and SF-12 from enrollment to one-year follow-up within each cohort because the distributions did not follow a normal distribution. Two sample *t*-tests were performed to compare GUPI and SF-12 scores between cohorts. General linear regression was conducted to assess the associations of patient demographic and clinical characteristics with GUPI and SF-12 total scores at baseline and with the score changes after one-year follow-up. All the analyses were performed using SAS 9.4 (SAS Institution Inc., Cary NC). All *p* values reported were 2-sided. A *p* value < 0.05 was considered statistically significant.

## Results

We enrolled 308 (78%) IC/BPS patients and 85 (22%) patients with OPPCs who completed both the GUPI and SF-12 at baseline. There was no statistical difference in demographic or clinical characteristics at enrollment between the IC/BPS cohort and the OPPC cohort (Table 1). The one-year follow-up GUPI and SF-12 were completed by 183 (59%) and 196 (64%) patients in the IC/BPS cohort, and by 65 (76%) and 62 (73%) patients in the OPPC cohort, respectively (Table 2).

**Table 1:** Demographic and clinical characteristics of patients with IC/BPS and OPPC.

	IC/BPS (*N* = 308)	OPPC (*N* = 85)	Total (*N* = 393)	*p*-value^1^
Age (year), mean (SD)	58 (14)	58 (14)	58 (14)	0.980
Gender, *n* (%)				0.180
Female	145 (47)	47 (55)	192 (49)	
Male	163 (53)	38 (45)	201 (51)	
Race, *n* (%)				0.292
Black	60 (19)	21 (25)	81 (21)	
Non-Black	248 (81)	64 (75)	312 (79)	
Non-Hispanic or Latino^2^, *n* (%)	274 (93)	81 (96)	355 (94)	0.275
History of depression, *n* (%)	118 (38)	35 (41)	153 (39)	0.632
History of alcohol abuse, *n* (%)	30 (10)	7 (8)	37 (9)	0.674
History of PTSD, *n* (%)	113 (37)	30 (35)	143 (36)	0.813
History of IBS, *n* (%)	70 (23)	13 (15)	83 (21)	0.137
Years from independent diagnosis to first questionnaire completion, mean (SD)	8 (5)	2 (2)	8 (5)	0.125

BPS, Bladder pain syndrome; IBS, Irritable bowel syndrome; IC, Interstitial cystitis; OPPC, Other pelvic pain conditions; PTSD, Post traumatic stress disorder.

^1^Chi-squared test for categorical variables and *t*-test for continuous variables.

^2^Missing values were excluded in calculation.

**Table 2:** Summary of GUPI and SF-12 total and sub-scores at enrollment and at one-year follow-up.

	IC/BPS				OPPC				*p*-value from t-test to compare IC/BPS and OPPC		
	Baseline	Follow-up	Change from baseline	*p*-value^1^	Baseline	Follow-up	Change from baseline	*p*-value^1^	Baseline	Follow-up	Change
GUPI, N	308	183	183		85	65	65				
	Mean (SD)^2^	Mean (SD)^2^	Mean (SD)^3^	** **	Mean (SD)^2^	Mean (SD)^2^	Mean (SD)^3^	** **	** **	** **	** **
Pain Symptom	12.5 (6.1)	14.0 (5.9)	**1.1 (4.5)**	**0**.**002**	16.6 (6.1)	18.1 (5.4)	**1.2** (**3.9)**	**0**.**022**	**<**.**001**	**<**.**001**	0.806
Urinary Symptom	4.1 (2.9)	4.8 (2.9)	**0.5 (2.4)**	**0**.**009**	6.1 (2.9)	6.5 (2.5)	0.3 (2.3)	0.303	**<**.**001**	**<**.**001**	0.624
QoL sub-score	4.8 (3.3)	5.4 (3.2)	**0.6 (2.4)**	**<**.**001**	7.0 (3.5)	7.7 (3.2)	**0.6** (**2.4)**	**0**.**039**	**<**.**001**	**<**.**001**	0.952
Total	21.4 (10.9)	24.3 (10.5)	**2.1 (7.5)**	**<**.**001**	29.8 (11.2)	32.4 (9.9)	**2.1** (**7.1)**	**0**.**018**	**<**.**001**	**<**.**001**	0.993
SF-12, N	308	196	196		85	62	62		** **	** **	
	Mean (SD)^2^	Mean (SD)^2^	Mean (SD)^3^		Mean (SD)^2^	Mean (SD)^2^	Mean (SD)^3^		** **	** **	
Physical Health	38.7 (11.0)	38.2 (9.5)	−0.3 (8.8)	0.722	41.6 (12.0)	40.8 (11.4)	−1.3 (7.3)	0.238	**0**.**039**	0.072	0.422
Mental Health	42.6 (12.8)	44.1 (11.9)	0.7 (10.5)	0.612	46.3 (11.4)	43.9 (12.2)	−**2.2** (**8.3)**	**0**.**043**	**0**.**015**	0.902	**0**.**046**
Total	81.3 (16.6)	82.3 (14.9)	0.4 (11.0)	0.413	87.9 (16.8)	84.7 (17.9)	−**3.5** (**10.8)**	**0**.**027**	**0**.**001**	0.294	**0**.**015**

Bolded items are statistically significant (*p* < 0.05).

BPS, Bladder pain syndrome; IC, Interstitial cystitis; GUPI, Genitourinary Pain Index; OPPC, Other pelvic pain conditions; SF-12, 12-item Short Form Health Survey.

^1^ Wilcoxon Signed-rank test to test the score change at one-year follow-up from baseline within each cohort.

^2^ A higher number means better health condition.

^3^ A positive number indicates health condition improved from baseline.

At baseline, IC/BPS cohort reported a mean pain symptom sub-score, urinary symptom sub-score, urologic HRQOL sub-score, and total GUPI score that were lower than baseline scores for OPPC, indicating that the IC/BPS cohort had worse mean values for urologic symptoms and HRQOL at baseline (*p* < 0.001, Table 2). At baseline, IC/BPS patients had significantly lower GUPI scores (8.4 points less than OPPC, *p* < 0.001) and lower SF-12 scores (6.6 points less than OPPC, *p* < 0.001). IC/BPC patients reported lower (worse) mean scores on the SF-12 physical health sub-score, mental health sub-score, and SF-12 total score than patients with OPPCs (Table 2).

At one year follow-up, the IC/BPS cohort had mean pain symptom sub-scores, urinary symptom sub-scores, urologic HRQOL sub-scores and total GUPI scores that remained lower than the OPPC cohort's corresponding scores. At follow-up, the mental health sub-scores, physical health sub-scores, and overall SF-12 mean scores were not significantly different between groups.

The change in GUPI scores from baseline to one-year follow-up showed that IC/BPS patients had improved pain symptom score by 1.1 (*p* = 0.002), improved urinary symptom score by 0.5 (*p* = 0.009), improved urologic HRQOL score by 0.6 (*p* < 0.001), and an improved GUPI total score by 2.1 (*p* < 0.001). In OPPC patients, there was no statistically significant change in the urinary symptom sub-score (*p* = 0.303) but there was improvement in the pain symptom sub-score by 1.2 (*p* = 0.022), improved urologic HRQOL sub-score by 0.6 (*p* = 0.039), and improved GUPI total score by 2.1 (*p* = 0.018) (Table 2). The change in total GUPI scores was comparable between the groups (0.0 points average difference between cohorts, *p* > .999) indicating both cohorts showed equivalent improvement during the study duration.

In analyzing these changes, IC/BPS patients showed no significant change in physical health, mental health, or total SF-12 score. In OPPC patients, the physical health sub-score remained unchanged (*p* = 0.238), but the mental health sub-score and SF-12 total score showed significant decreases (worsening) in scores at follow-up. Despite IC/BPS having significantly worse general HRQOL at baseline (*p* = 0.001), the follow-up SF-12 score showed no difference in general HRQOL between the two cohorts (*p* = 0.294) after one-year because the OPPC patient's general HRQOL deteriorated. IC/BPS patients showed significantly higher improvements in general HRQOL over the study duration (4.1 points greater change in SF-12, *p* = 0.012). In summary, while IC/BPS had worse health at baseline, their scores remained unchanged in all general HRQOL measured by the SF-12. Meanwhile, OPPC patients showed deteriorating mental health and general HRQOL.

In the IC/BPS cohort the baseline GUPI scores were impacted by a patient's age (0.2 points higher per year older, *p* < .001) and history of PTSD (3.5 points lower, *p* = 0.014) while SF-12 baseline was impacted by a patient's age (0.2 points higher per year older, *p* = 0.003), race being African American/black (3.7 points lower, *p* = 0.094), and a history of depression (7.4 points lower, *p* < .001) (Table 3). None of these parameters impacted the change from baseline.

**Table 3:** Associations of IC/BPS patient characteristics with GUPI and SF-12 total scores at baseline and with the score change after 1-year follow-up.

Parameter	GUPI				SF-12			
	Baseline		Change from baseline		Baseline		Change from baseline	
	Mean (SE)	*p*-value	Mean (SE)	*p*-value	Mean (SE)	*p*-value	Mean (SE)	*p*-value
Age (per 1 year)	**0.2** **(0.0)**	**<**.**001**	0.1 (0.1)	0.120	**0.2** (**0.1)**	**0**.**003**	0.0 (0.1)	0.575
Male (ref = female)	0.4 (1.3)	0.764	−0.5 (1.4)	0.732	0.1 (2.0)	0.958	−3.3 (1.9)	0.093
Black (ref = non-White)	−1.4 (1.5)	0.342	−0.8 (1.6)	0.619	−**3.7** (**2.2)**	**0**.**094**	−0.1 (2.2)	0.978
History of depression (ref = no depression)	−0.4 (1.3)	0.792	1.2 (1.3)	0.375	−**7.4** (**2.0)**	**<**.**001**	0.1 (1.9)	0.949
History of alcohol abuse (ref = no alcohol abuse)	0.4 (2.0)	0.830	1.6 (2.0)	0.423	−1.5 (3.0)	0.624	2.6 (2.9)	0.376
History of PTSD (ref = no PTSD)	−**3.5** (**1.4)**	**0**.**014**	0.6 (1.4)	0.669	−2.8 (2.1)	0.178	1.1 (2.0)	0.573
History of IBS (ref = no IBS)	−2.1 (1.4)	0.155	0.8 (1.4)	0.576	−4.2 (2.2)	0.054	−0.1 (2.0)	0.957

Bolded items are statistically significant (*p* < 0.05).

Higher scores are indicative of better health. General linear model was fit to get the estimated mean, standard error (SE), and *p*-value.

BPS, Bladder pain syndrome; IBS, Irritable bowel syndrome; IC, Interstitial cystitis; GUPI, Genitourinary Pain Index; PTSD, Post traumatic stress disorder; SE, Standard error; SF-12, 12-item Short Form Health Survey.

In the OPPC cohort, the baseline GUPI scores were impacted by a patient's race being African American/black (10 points lower, *p* < .001) and a history of irritable bowel syndrome (IBS) (8.2 points lower, *p* = 0.015) while SF-12 baseline scores were impacted by a history of depression (11 points lower, *p* = 0.005) and history of PTSD (8.3 points lower, *p* = 0.048) (Table 4). Only age impacted the change from baseline on the GUPI (0.2 points improvement per year older, *p* = 0.021).

**Table 4:** Associations of OPPC patient characteristics with GUPI and SF-12 total scores at baseline and with the score change after 1-year follow-up.

Parameter	GUPI				SF-12			
	Baseline		Change from baseline		Baseline		Change from baseline	
	Mean (SE)	*p*-value	Mean (SE)	*p*-value	Mean (SE)	*p*-value	Mean (SE)	*p*-value
Age (per 1 year)	−0.1 (0.1)	0.363	**0.2** **(0.1)**	**0**.**021**	−0.0 (0.2)	0.763	0.1 (0.1)	0.581
Male (ref = female)	−0.1 (2.8)	0.969	−2.3 (2.3)	0.335	−0.2 (4.3)	0.954	−0.8 (3.8)	0.833
Black (ref = non-White)	−**10** (**2.6)**	**<**.**001**	0.8 (2.3)	0.736	−3.3 (4.1)	0.417	3.5 (3.9)	0.372
History of depression (ref = no depression)	−4.7 (2.5)	0.064	2.2 (2.0)	0.282	−**11** (**3.9)**	**0**.**005**	−1.3 (3.3)	0.690
History of alcohol abuse (ref = no alcohol abuse)	−5.9 (4.3)	0.172	2.2 (3.8)	0.559	−2.4 (6.6)	0.716	8.0 (6.2)	0.203
History of PTSD (ref = no PTSD)	−3.4 (2.7)	0.209	−0.2 (2.1)	0.933	−**8.3** (**4.1)**	**0**.**048**	−0.4 (3.4)	0.911
History of IBS (ref = no IBS)	−**8.2** (**3.3)**	**0**.**015**	2.8 (2.7)	0.311	−3.1 (5.1)	0.553	−0.2 (4.6)	0.967

Bolded items are statistically significant (*p* < 0.05).

Higher scores are indicative of better health. General linear model was fit to get the estimated mean, standard error (SE), and *p*-value.

IBS, Irritable bowel syndrome; GUPI, Genitourinary pain index; OPPC, Other Pelvic Pain Conditions; PTSD, Post traumatic stress disorder; SE, Standard error; SF-12, 12-item short form health survey.

## Discussion

We had several key findings from this cohort study of heterogeneous patients seeking care in the VA system. The IC/BPS cohort had significantly worse scores for all baseline sub-scores of the GUPI (*p* < 0.001), suggesting that IC causes significantly worse pelvic pain than other pelvic conditions. Despite comparable improvements on the GUPI in both cohorts, we found that IC/BPS scores at follow-up were also significantly worse in every sub-score of the GUPI (*p* < 0.001) further supporting this conclusion that IC/BPS causes worse pain and poses a greater impact on urologic HRQOL than other pelvic conditions. Thus, during the treatment of IC/BPS, additional care towards urinary pain and management is needed to address the significant gap observed in urologic HRQOL.

Despite worse pain, the IC/BPS cohort showed significant improvements in all measures of the GUPI from baseline to follow-up. Though this showed improving urinary conditions, the SF-12 showed no change in any sub-score or total score. Since SF-12 represents general HRQOL, the combined impact on physical and mental health, these results suggest that IC/BPS has more of a condition-specific impact on HRQOL than a general HRQOL impact. The MAPP network has previously studied HRQOL in IC/BPS and chronic prostatitis/chronic pelvic pain syndrome (CP/CPPS) and found that significantly more patients experienced urinary-specific HRQOL improvements at follow-up than general HRQOL (4). In this study, 47% of the patients had improved urologic specific outcomes, while urologic outcomes were stable or worse in 53%. The same cohort showed only 25% improvement in general HRQOL measures, while 75% showed stable or worse general HRQOL outcomes. Similar to our study, the largest benefit at follow-up was seen in urologic specific outcomes, with majority showing no change in general HRQOL for IC/BPS patients.

In the OPPC cohort, the GUPI improved in all sub-measures except urinary symptoms, while still maintaining an improvement in total GUPI score. In this cohort, SF-12 mental health sub-scores and total SF-12 deteriorated over the span of the study. This shows that, despite improvement in urologic HRQOL and management of urinary pain, the patient's general HRQOL still deteriorated. The absence of urinary symptom improvement might have caused worsening HRQOL as patients struggle to see improvements in aspects of their condition causing greater stresses on their general health. The MAPP Network also noted that worsening general HRQOL measures were indicative of more widespread non-urologic pain and discomforts (4). This might also be another potential cause in the deterioration in the OPPC as these conditions might pose more widespread symptoms with non-urologic impacts on HRQOL.

Comparing the two cohorts, both IC/BPS and OPPC demonstrated improvements from baseline in urologic HRQOL over a one-year follow-up, and the improvements were to the same degree, showing no difference in the change in total GUPI score. This might be due to receiving similar treatments for pelvic pain in both cohorts, but these modalities were not tracked in this study. When compared, IC/BPS patients showed 4.1 times the improvement in general HRQOL compared to the OPPC cohort, showing the significant impact that OPPC conditions have on deteriorating general health. A patient's physical and mental health needs to be a primary concern in the treatment of patients with these pelvic conditions, considering that mental health was the most significantly impacted measure at follow-up. Depression significantly impacted the SF-12 in both cohorts. Given that depression co-occurs with pelvic pain at a high frequency, mental health professionals should be included in the treatment process of these individuals to not only treat the condition but ensure a holistic approach to patients' QOL. A study from Brazil found that women with pelvic pain had a prevalence rate of 73% for anxiety and 40% for depression, significantly higher than among controls (8). Multiple studies have also shown an independent association between a patient's pelvic pain diagnosis and developing depression, anxiety, and other mental illnesses (8–10). It might also be necessary for the incorporation of more holistic treatments in OPPC conditions, since general HRQOL deteriorated over our study.

Older individuals with IC/BPS demonstrated higher baseline GUPI and SF-12 indicating better urinary and general HRQOL with age. One of the primary interventions for IC/BPS remains lifestyle and behavioral modifications that help patient's cope with their symptoms. Our data might suggest that these older patients have been able to positively cope with symptoms and create a positive impact on their HRQOL through these measures. Previous studies by the MAPP Network have looked at both duration of symptoms and age as effects on pain severity and symptoms and found that females better coped with advancing age, regardless of duration of symptoms (11). In fact, similar studies have shown similar results in regards to aging, showing improvements in the SF-12 questionnaire as IC/BPS patients age (12, 13). Both improved coping skills with increasing age and more established diagnosis and treatment over time are likely the cause of this improvement with age.

Our study also showed that compared with patients of other races, black patients had worse baseline SF-12 scores in the IC/BPS cohort and worse baseline GUPI scores in the OPPC cohort. A previous study noted that IC/BPS symptoms are equally common in black and white individuals (3), suggesting that while they have similar frequency of symptoms, minorities might shoulder a larger burden as we observed significantly lower baseline scores for black patients in this study. Additionally, the Boston Area Community Health (BACH) Survey looked at a cohort of men and women to estimate the prevalence of symptoms suggestive of Painful Bladder Syndrome (PBS) and associations with socio-demographic features (14). This study found that the prevalence of symptoms suggestive of PBS was greater among minorities (black and Hispanic) and in those with lower socio-economic status. Our results support and demonstrate that racial minorities (black) might bear a larger burden in these pelvic conditions with potential impacts to urological HRQOL and/or general HRQOL as seen in lower baseline HRQOL scores.

History of other physical and mental ailments also impacted baseline scores in both cohorts. History of depression in both IC/BPS patients and OPPC patients showed significantly lower SF-12 scores—general HRQOL—at baseline. Previous studies looking at pelvic pain demonstrated that depression is independently associated with lower HRQOL and worse outcomes (4), supporting our findings. PTSD negatively impacted baseline GUPI scores for IC/BPS patients and SF-12 scores for OPPC patients. A previous study showed that the presence of non-pelvic conditions including IBS in IC/BPS patients led to detrimental impacts on HRQOL. They found significantly higher Interstitial Cystitis Symptom Index scores (showing greater symptom effect) and significantly lower SF-36 scores (showing lower general HRQOL) (15). IBS also contributed to lower baseline GUPI scores in the OPPC cohort. The MAPP network has found that patients with IC/BPS with other nonurological associated syndromes including IBS reported more severe pain, worse physical quality of life, more depression, stress, and sexual dysfunction than patients who do not have IBS (16). These impacts have been shown to decrease quality of life in these patients which was observed in this study.

Our study is unique in providing a cohort evenly matched for both race and gender. Historically, IC has been noted as an illness for white females (17–19), but more recent studies have begun to show that minorities might shoulder a larger burden than previously thought (3). Our study addresses this gap by enrolling a diverse and matched study population for various demographics (Table 1), something unique from the literature. As a treated natural history study, participants received usual care for their conditions over the study duration. The improvements in GUPI components in both cohorts may in part reflect these treatments. We previously conducted a retrospective analysis of women with confirmed IC/BPS and those with OPPC using the same cohort definitions to identify prescription rates of nonnarcotic pain medications used in treating these conditions (20). This analysis found that IC/BPS patients were given nonnarcotic bladder pain medication more often than those with OPPCs. Despite this fact and observing that over 77% of women with IC/BPS in the study received treatment, we found that IC/BPS patients had a significant increase in the number of medications used and required multiple prescriptions for pain, highlighting the difficulty in effectively treating IC/BPS (20). These treatment findings help support the conclusion that IC/BPS have worse urologic pain compared to OPPCs as demonstrated on the GUPI. While the current study included both men and women, the review of prescription practices included only women who met the same cohort criteria and further work is needed to determine if the same prescription practices are seen in men. Individual treatment details were not tracked as part of this analysis, given the heterogeneous nature of treatments and limited resources available for manual chart abstraction in this observational study. Our study focused on the pain and HRQOL outcomes faced by these two populations after diagnosis and demographic details were independently confirmed by chart review. Further studies are needed into tracking treatment modalities and correlating it to the effected change in HRQOL over time. Since individual treatments, outcomes and progression were not followed during the progression, it is possible that those who did not respond to questionnaires at follow-up could have different responses from the observed population over time which should remain a limitation of this study. Future studies are also needed to further evaluate the impact of these pelvic conditions on racial and ethnic minorities as studies have suggested they might shoulder a larger burden.

## Conclusion

In a heterogenous population of veterans nationwide we found that patients with IC/BPS had worse urologic pain compared to patients with other pelvic conditions. We also found that IC/BPS had no change in general HRQOL compared with deteriorating general HRQOL in OPPC over a one-year period. This suggests that IC/BPS has more of a condition-specific impact on urologic health while OPPC conditions may have extensive impacts on general health due to wider non-urologic symptoms. Additional research with longer follow-up is needed to better understand these factors and IC's disease specific burden on HRQOL.

## Data Availability

The raw data supporting the conclusions of this article will be made available by the authors, without undue reservation.

## Appendix 1. Summary of ICD Codes utilized in cohort identification

**Table:**

	ICD-9	ICD-10
Interstitial Cystitis	595.1	N30.10, N30.11
Chronic Prostatitis	601.1	N41.1
Dyspareunia	625.0	N94.1
Vaginismus	625.1	N94.2
Vulvar Vestibulitis	625.71	N94.810
Vulvodynia	625.70	N94.819
